# Base Station Placement Algorithm for Large-Scale LTE Heterogeneous Networks

**DOI:** 10.1371/journal.pone.0139190

**Published:** 2015-10-13

**Authors:** Seungseob Lee, SuKyoung Lee, Kyungsoo Kim, Yoon Hyuk Kim

**Affiliations:** 1 Dept. of Computer Science, Yonsei University, Seoul, Republic of Korea; 2 Dept. of Applied Mathematics, Kyung Hee University, Yongin, Republic of Korea; 3 Dept. of Mechanical Engineering, Kyung Hee University, Yongin, Republic of Korea; Universitat Rovira i Virgili, SPAIN

## Abstract

Data traffic demands in cellular networks today are increasing at an exponential rate, giving rise to the development of heterogeneous networks (HetNets), in which small cells complement traditional macro cells by extending coverage to indoor areas. However, the deployment of small cells as parts of HetNets creates a key challenge for operators’ careful network planning. In particular, massive and unplanned deployment of base stations can cause high interference, resulting in highly degrading network performance. Although different mathematical modeling and optimization methods have been used to approach various problems related to this issue, most traditional network planning models are ill-equipped to deal with HetNet-specific characteristics due to their focus on classical cellular network designs. Furthermore, increased wireless data demands have driven mobile operators to roll out large-scale networks of small long term evolution (LTE) cells. Therefore, in this paper, we aim to derive an optimum network planning algorithm for large-scale LTE HetNets. Recently, attempts have been made to apply evolutionary algorithms (EAs) to the field of radio network planning, since they are characterized as global optimization methods. Yet, EA performance often deteriorates rapidly with the growth of search space dimensionality. To overcome this limitation when designing optimum network deployments for large-scale LTE HetNets, we attempt to decompose the problem and tackle its subcomponents individually. Particularly noting that some HetNet cells have strong correlations due to inter-cell interference, we propose a correlation grouping approach in which cells are grouped together according to their mutual interference. Both the simulation and analytical results indicate that the proposed solution outperforms the random-grouping based EA as well as an EA that detects interacting variables by monitoring the changes in the objective function algorithm in terms of system throughput performance.

## 1 Introduction

The last two decades have witnessed a boom in the use of cellular communication technologies. Billions of people are now requesting high-quality mobile wireless services with end-user data rates of several megabits per second over wide areas and tens, or even hundreds end is expected to continue in the future, with volumes predicted to increase around 15 times their current levels by 2016—2017, reaching 11.2 exabytes per month by 2017 [1, 2]. Recently, in order to meet and encourage such ever-increasing service demands, heterogeneous networks (HetNets) have been widely discussed, perhaps most significantly in the 3GPP long term evolution-advanced (LTE-A). In HetNets, small cells complement traditional macro eNodeB (eNB) cells by extending coverage to indoor areas which outdoor signals have difficulty reaching, or by increasing network capacity in areas of highly dense phone usage such as train stations, airports, and shopping malls. However, deploying small cells as parts of HetNets creates a key challenge for operators’ careful network planning. HetNets are becoming increasingly complex due to the deployment of heterogeneous cells that have distinctly different traits. In particular, HetNets show large degrees of variation in both the number of interfering cells and in the amount of interference. To make matters worse, massive and unplanned deployment of these base stations (BSs) cause a much higher magnitude of interference, potentially resulting in highly degraded network performance.

In conventional cellular networks, a large amount of prior work has dealt with the planning and optimization of cellular access network design and operations, with problem formulations including coverage planning, power optimization, and channel assignment [3, 4]. Although different mathematical modeling and optimization methods have been used to approach these problems, most traditional network planning models are ill-equipped to deal with HetNet-specific characteristics due to their focus on classical cellular network design. Furthermore, increased wireless data demands have driven mobile operators to roll out large-scale networks of small LTE cells. For instance, Sprint has planned to make aggressive use of small cells in its future LTE network, launching tens of thousands of tiny high-capacity BSs in high-traffic indoor and outdoor areas in 2013 and 2014 [5]. Therefore, we aim to derive an optimum network planning algorithm for the large-scale LTE HetNets.

In 3G cellular networks, mobile operators carefully try to choose the locations of new BSs in order to meet increasing demands for wireless coverage and larger data rates. A large amount of previous work has focused on how to locate and configure new macro BSs. One of the first BS placement algorithms was presented by Sherali et al. [3], who considered single and multiple transmitter problems. In [6], a genetic approach was used to find the near-optimal locations of BSs. Amaldi et al. [7] proposed discrete optimization algorithms to support decisions in choosing the locations of new BSs from a set of candidate sites, considering signal quality constraints in the uplink direction and fixed BS configuration. They also considered the downlink direction, since 3G systems are specifically intended to provide data services for users [8]. However, in practice, mobile operators usually have a tightly limited set of candidate sites due to authorization constraints on new antenna installation, and site acquisition costs are very expensive in urban areas. Thus, they investigated mathematical programming models for 3G radio planning, given that modifying the configuration of existing BSs can also provide improved wireless coverage for users [9]. In [10] and [11], mixed integer linear programming was employed for planning cost-efficient radio networks under network quality constraints. Models based on set covering were used to obtain lower bounds on the number of BSs required to serve a given fixed area, and an automatic two-phase network planning approach based on successive instances of model application was presented. In [12], a net-revenue maximization model for the selection of BS sites and the calculation of service capacity was presented. Recently, addressing the point that the majority of contributions to optimized network planning have focused on the selection of a minimal BS set from a larger candidate fixed BS set, Khalek et al. [4] presented optimization-based formulations for the problems of joint uplink/downlink site placement and site selection in cellular networks. In the site-selection algorithm, the minimum set of BSs is selected from a fixed set of candidate sites that satisfy quality and outage constraints. The placement of BSs is then determined in a subset of the deployment area according to private property limitations or electromagnetic radiation constraints. However, while the site-selection and site-placement algorithms provide a more locally optimal solution with a lower number of BS, a larger number of BSs requires more computation time.

Because evolutionary algorithms (EAs) can be characterized as global optimization methods, they have been utilized successfully in a variety of complicated real-world applications [13]. Several methods have also been developed spontaneously in the field of radio network planning, mainly based on EAs [14–16]. Weicker et al. [14] proposed a steady-state EA with Pareto tournaments (stEAPT), which considers frequency assignment and channel interference for BS placement. Most recently, addressing the observation that multiple objectives (MO) must be taken into account when solving the wireless heterogeneous transmitter placement problem, Ting et al. [15] proposed to integrate a novel variable-length representation and a new crossover approach into their non-dominated sorting genetic algorithm II (NSGA II) [16], which is known for its effectiveness in dealing with MO problems. However, one crucial difficulty in employing EAs is the huge time consumption resulting from the high complexity of performance analyses for fitness evaluation and the large number of evaluations needed in evolutionary optimization techniques. Accordingly, the performance of EAs often deteriorates rapidly with the growth of search space dimensionality.

To overcome the problems mentioned above when designing optimum network deployments for large-scale LTE HetNets, we attempt to decompose the high-dimensionality problem and tackle its subcomponents individually. We propose a grouping method to divide the candidate solutions (individuals) in the populations into groups. Noting that some HetNet cells have strong correlations due to inter-cell interference, we propose to use a correlation grouping approach instead of grouping the individuals randomly with the aim of rapidly converging to optimal solutions. In this approach, variables with strong correlations (i.e., interfering cells) form a group when finding the optimal deployment of heterogeneous cells in the HetNet. In addition, we modify the variable-length genetic algorithm presented in [15] to be applied to the divided groups.

The rest of this paper is organized as follows: In Section 2, we include a short review of how EAs and their variants have been applied for solving the problems in various applications including the BS placement problem. Section 3 presents the mathematical formulation of the BS deployment problem optimization in the LTE HetNet. Section 4 presents the proposed grouping method and the strategy for solving the optimization problem based on the variable-length genetic algorithm [15]. In Section 5, we provide an analytical model of the probability of two BSs placed in the same group interfering with each other, as well as the corresponding numerical results, and report simulation results for different user distributions. Finally, conclusions are drawn in Section 6.

## 2 Related Work

EAs are characterized as global optimization methods and are generally known to be robust optimizers that are well suited for objective functions that are discontinuous and have many non-smooth changes [17, 18]. For this reason, they have been applied successfully to a variety of complicated real-world applications such as discovery of link communities in complex networks [13], financial and economic applications [19], aircraft conflict avoidance [20, 21], demand side management in smart grids [22], etc.

The BS placement problem is to find the optimal positions of BSs, considering various controlled and uncontrolled factors of traffic density, capacity, interference, existing BSs, etc. Due to the combined effects of these factors, the problem cannot be solved in polynomial time; that is known to be NP-hard [4]. Some heuristic methods based on the evolutionary paradigm have also been developed for finding high-quality solutions to such BS placement problems [14, 15]. In [14], the steady-state EA with Pareto tournaments (stEAPT) approach is introduced as a new MO technique that considers frequency assignment and channel interference for BS placement. This approach combines a steady-state scheme with a very efficient data structure leading to superior time complexity. Most recently, addressing the observation that MO must be taken into account when solving the wireless heterogeneous transmitter placement problem, Ting et al. [15] proposed to integrate a novel variable-length representation and a new crossover approach into their non-dominated sorting genetic algorithm II (NSGA II) [16], which is known for its effectiveness in dealing with MO problems. However, one crucial difficulty in employing EAs is the huge time consumption resulting from the high complexity of performance analyses for fitness evaluation and the large number of evaluations needed in evolutionary optimization techniques. Accordingly, the performance of EAs deteriorates rapidly with the growth of search space dimensionality. Apparently, this is also the case with the BS placement problem in LTE HetNets because explosive mobile data demands have driven mobile operators to deploy LTE small cells on a large scale.

Cooperative co-evolution (CC) has been introduced into EAs with the aim of solving increasingly large and complex optimization problems through a divide-and-conquer paradigm [23]. Nonetheless, existing CC algorithms did not take into account variable interdependencies for nonseparable problems in which tight interactions exist among different decision variables. To efficiently tackle nonseparable problems, some CC frameworks were proposed relying on random grouping that randomly allocates the variables to subcomponents in every co-evolutionary cycle [24–26], instead of using a static grouping. These algorithms do not provide a systematic procedure to group the interacting variables nor to detect their interdependencies, even though it was shown in [24] that with random grouping, the probability of placing two interacting variables in the same subcomponent for several cycles is reasonably high.

More recently, some algorithms have been proposed to identify and group interacting variables into common subcomponents in various real-world optimization problems [20, 21, 23]. In [20] and [21], CC frameworks with dynamic grouping strategies were proposed to guarantee safety in air traffic control. In the dynamic grouping strategy, a large number of aircraft are divided into several sub-groups based on their interdependence and the sub-groups are adjusted dynamically as new conflicts appear after each iteration. Omidvar et al. [23] proposed a decomposition method called differential grouping that is able to group the interacting variables with high accuracy, focusing on large-scale global optimization problems. Here, it should be noted that these algorithms focus on discovering the interdependencies between variables, whereas in the LTE HetNets, the effect of inter-cell interference should be designed as various levels of interdependence, since the interference is one of the most critical factors to be considered when deploying small cells. Specifically, the degree of interdependence between cells varies based on the amount of inter-cell interference, not the existence of interdependence. Thus, we cannot efficiently group the cells with strong interdependencies by simply applying the existing grouping methods to the BS placement problem.

In this paper, we propose to use a correlation grouping approach with the aim of rapidly converging to optimal solutions. In this approach, variables with strong correlations (i.e., interfering cells) form a group when finding the optimal deployment of heterogeneous cells in the HetNet. In addition, we modify the variable-length genetic algorithm presented in [15] to be applied to the divided groups.

## 3 BS Deployment Optimization Problem Formulation

In this section, we present a formulation of the BS deployment optimization problem in the LTE HetNet, defining the objective, variables, and constraints for the problem. In this study, we are given a domain, *D*, in the HetNet that must be covered by heterogeneous BSs with different transmission powers. For the given domain, we aim to find a BS deployment plan that maximizes user satisfaction in terms of the throughput provided per unit of traffic demand from the user.

We assume that there are *M* BSs in the domain *D*, and that each BS is located at the position (*x*_*b*_, *y*_*b*_) ∈ *D* (1 ≤ *b* ≤ *M*) with the transmission power *p*_*b*_, where *M* is a constant value determined by the network operator. Let *p*_*max*_ be the maximum transmit power and 0 ≤ *p*_*b*_ ≤ *p*_*max*_. It is also assumed that there are *N* users and that each user is located at the position (*X*_*u*_, *Y*_*u*_) (1 ≤ *u* ≤ *N*) with a traffic demand, *d*_*u*_.

The signal-to-interference-plus-noise-ratio (SINR) at the *u*-th user is then given by

$$\begin{array}{c} S_{b,u}=\frac{p_{b}l_{b,u}}{N_{0}+\sum_{1\leq b^{\prime }\leq M,b^{\prime }\neq b}p_{b^{\prime }}l_{b^{\prime },u}}, \end{array}$$ (1)

where *N*_0_ is the noise power and *l*_*b*,*u*_ is the path loss between the BS, *b*, and the user, *u*, which is given by $l_{b,u}=10^{\frac{{\hat{l}}_{b,u}}{10}}$ for ${\hat{l}}_{b,u}=128.1+37.6\;\text{log}\sqrt{{\left(x_{b}-X_{u}\right)}^{2}+{\left(y_{b}-Y_{u}\right)}^{2}}$ (in dB) [27].

We denote with *T*_*u*_ and *U*_*u*_ the LTE downlink throughput and the user satisfaction for *u*-th user, respectively. Given that the *u*-th user is served by the *b*-th BS, we can express *T*_*u*_ and *U*_*u*_ as follows:

$$\begin{array}{c} T_{u}=C\times R_{u}\times \log_{2}(1+S_{b,u}) \end{array}$$ (2)

and

$$\begin{array}{c} U_{u}=\min(\frac{T_{u}}{d_{u}},1), \end{array}$$ (3)

where *R*_*u*_ is the number of resource blocks (RBs) assigned to the *u*-th user, and *C* = 180*kHz* is the bandwidth of an RB [27].

For a given ordered set of triples containing the position and the traffic demand of every user,

$$\begin{array}{c} U=\{\left(X_{1},Y_{1},d_{1}\right),\cdots ,\left(X_{u},Y_{u},d_{u}\right),\cdots ,\left(X_{N},Y_{N},d_{N}\right)\}, \end{array}$$ (4)

the system satisfaction function *F*_*U*_ is defined as

$$\begin{array}{c} F_{U}\left(B\right)=\sum_{1\leq u\leq N}U_{u} \end{array}$$ (5)

for the ordered set of triples containing the position and transmit power of every BS,

$$\begin{array}{c} B=\{\left(x_{1},y_{1},p_{1}\right),\cdots ,\left(x_{b},y_{b},p_{b}\right),\cdots ,\left(x_{M},y_{M},p_{M}\right)\}. \end{array}$$ (6)

Let *A* be the set of all possible BS deployments in the domain *D* with a maximum transmission power of *p*_*max*_, and let *H*_*b*_ be the set of all users served by the *b*-th BS. The problem of finding the optimal BS deployment in *A* is then formulated as follows:

$$\begin{array}{c} {\text{Maximize}}_{B\in A}F_{U}\left(B\right) \end{array}$$ (7)

s.t.

$$\begin{array}{c} \sum_{u\in H_{b}}R_{u}\leq R_{max}\;\;\;\left(1\leq b\leq M\right), \end{array}$$ (8)

where *R*_*max*_ is the maximum number of RBs that can be allocated, being set to 50 when using a 20 MHz system bandwidth in LTE frequency division duplex (FDD) downlink [28].

## 4 Cooperative Co-evolution with BS Grouping

In this section, we describe the proposed grouping method and the EA used to solve the BS placement optimization problem.

### 4.1 BS Grouping Algorithm

As addressed in Section 1, increased wireless data demands have driven mobile operators to roll out large-scale networks of LTE small cells. Accordingly, the problem domain presented in the previous section is considered quite large, leading to an increased complexity in solving the problem. We note that cooperative co-evolution has been proposed to solve large and complex problems through problem decomposition [29]. Based on this notion, we decompose the BS placement optimization problem into subproblems by dividing all the BSs into different groups.

Let *B*_1_, *B*_2_, ⋯, *B*_*G*_ be disjoint subsets of *B*, where *B*_*j*_ = {(*x*_*b*_1__, *y*_*b*_1__, *p*_*b*_1__), ⋯, (*x*_*b*_*n*_*j*___, *y*_*b*_*n*_*j*___, *p*_*b*_*n*_*j*___)} (1 ≤ *j* ≤ *G*). Thus, *B* can be re-expressed as *B* = *B*_1_ ∪ *B*_2_ ∪ ⋯ ∪ *B*_*G*_. The subset *B*_*j*_ is obtained as follows:

1. *G* pivot points are randomly chosen in the domain *D*, each of which represents each group *B*_*j*_.
2. Each BS is included in a subset *B*_*j*_, in which the BS has the highest received signal strength among *G* disjoint subsets.

### 4.2 Proposed Evolutionary Algorithm

In this section, we present the details of our proposed EA for finding the optimal solution to the BS placement problem. Following the general concept of EAs, the algorithm starts with a population *P* composed of a set of individuals *B* ^(*i*)^. The proposed EA is iterated until the solution converges to the optimal placement. Each EA consists of the following four steps: fitness evaluation, parent selection, crossover and mutation.

#### 1) Fitness evaluation

At the beginning of each iteration, all the individual’s fitnesses are evaluated by computing the objective function (i.e., fitness function) in Eq (5). Thus, the higher the system throughput, the higher the fitness value. Then, only the best 50% are retained for the next generation.

#### 2) Parent Selection

For each of the groups generated by the BS grouping algorithm presented in Section 4.1, a pair of parents is selected for the next generation. Specifically, for each group in the individual *B* ^(*i*)^, $B_{j}^{\left(i\right)}$ itself becomes a parent, while the other parent $B_{j}^{\left(i^{\prime }\right)}$ (*i*′ ≠ *i*) is selected from the best 50% of individuals, excluding the parent already selected, with the probability *β*_*i*_, where

$$\begin{array}{c} \beta_{i}=\frac{F_{U}\left(B^{\left(i\right)}\right)}{\sum_{\forall B^{\left(i^{\prime }\right)}\in P,i^{\prime }\neq i}F_{U}\left(B^{\left(i^{\prime }\right)}\right)}. \end{array}$$ (9)

If the typical parent selection strategies available in the literature [14, 15] were applied to our EA, it would not be possible to decide which of its two parents a child belongs to. However, no such ambiguity exists in the parent selection process described above since only one child is generated for each individual.

#### 3) Crossover

A child individual is produced from the two selected parents through crossover and mutation. Given the crossover rate *P*_*c*_, the hybrid crossover has three ways of producing a child [15]: 1) perform only uniform crossover with the probability *P*_*c*_ × *P*_*c*_; 2) perform both uniform crossover and one-point crossover with the probability *P*_*c*_ × (1 − *P*_*c*_); or 3) perform only one-point crossover with the probability (1 − *P*_*c*_).

Regarding the representation of the position and transmission power of BS, *B* ^(*i*)^, (*x*_*i*_, *y*_*i*_, *p*_*i*_) consists of 24 bits, where the leftmost 16 bits (2 × 8 bits) and the rightmost 8 bits indicate the position and the transmission power, respectively. Then, we apply the uniform crossover method equiprobably to choose which of the two parents the child will inherit a bit from.

We now propose to modify the one-point crossover between two parents, $B_{j}^{\left(i\right)}$ and $B_{j}^{\left(i^{\prime }\right)}$. We denote by $|B_{j}^{\left(i\right)}|$ the number of tuples consisting of $B_{j}^{\left(i\right)}$, that is the number of BSs belonging to the set, $B_{j}^{\left(i\right)}$. Then, the proposed one-point crossover proceeds as follows:

Step 1) A random point is chosen in the range of $\left[0,\text{min}(|B_{j}^{\left(i\right)}|,|B_{j}^{\left(i^{\prime }\right)}|)\right]$. Each parent is divided into two parts at this point.
Step 2) A child is produced by combining the first part of $B_{j}^{\left(i\right)}$ with the second part of $B_{j}^{\left(i^{\prime }\right)}$, which contains the same number of tuples as the second part of $B_{j}^{\left(i\right)}$. If $|B_{j}^{\left(i\right)}|>|B_{j}^{\left(i^{\prime }\right)}|$, then the $\left(|B_{j}^{\left(i^{\prime }\right)}|+1\right)$-th to $|B_{j}^{\left(i\right)}|$-th tuples of $B_{j}^{\left(i\right)}$ are combined with the child generated in this step.

**Fig 1:**
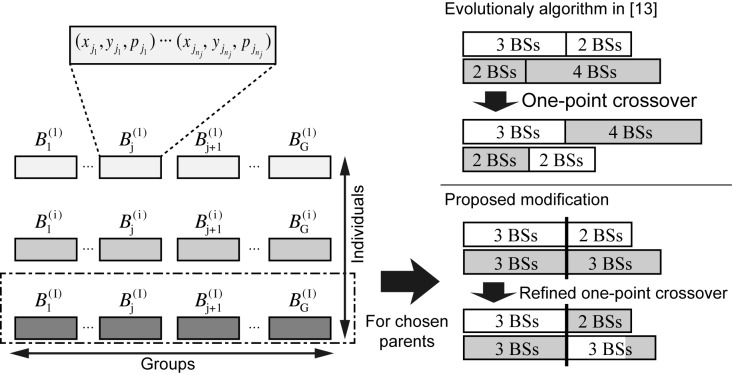
Examples of the one-point crossover presented in [15] and the refined one-point crossover.

Under the assumption made regarding *M*, the total number of BSs, *M*, in the domain, *D*, is constant. However, while the length of a child is variable in [15], leading to a value different from *M*, the one-point crossover proposed here maintains the length of the individual to be *M* over subsequent generations. Examples of the one-point crossover presented in [15] and the proposed method are depicted in Fig 1. It can be seen in Fig 1 that the two children have different lengths (7 and 4 tuples) from those of Parent 1 and Parent 2 (5 and 6 tuples, respectively) in the one-point crossover [15], while the proposed EA keeps the length of the child the same as that of its parent.

#### 4) Mutation

After the crossover, a bit-flip mutation with the mutation rate *P*_*m*_ is performed, giving each of the child’s bits a chance to flip.

## 5 Results

As explained in the Section 1, the proposed correlation grouping approach, which enables interfering cells to be placed together in one group, is a key contribution, since some cells have a strong correlation due to inter-cell interference in the HetNet. Thus, in this section, the probability of placing two interacting variables into a single group is first analyzed numerically for our proposed approach and a random grouping scheme [24–26]. We then provide evidence, based on simulation results, in support of throughput performance improvement of the proposed scheme over the random grouping scheme.

### 5.1 Probability of Interacting Variables Belonging to the Same Group

We denote by *P*_*g*_ the probability of two BSs being placed together in a single group at least *N*_*k*_ times during *N*_*c*_ cycles, where a cycle consists of one complete evolution of all groups. We simply refer to this probability as the *grouping probability* in this paper. In the random grouping scheme, the grouping probability *P*_*g*_ is derived as follows [24–26]:

Pg=∑r=NkNc(Ncr)(1G)r(1-1G)Nc-r. (10)

Note that the SINR is subjected to considerable attenuation over distance between the transmitter and the receiver as seen in Eq (1). Thus, we derive the grouping probability in our proposed EA based on the distance between BSs. We assume that BSs are distributed over the domain *D* according to a Poisson point process (PPP) with mean density, $\lambda =\frac{M}{A(D)}$, where *A*(*D*) denotes the area of the domain *D*. It is also assumed that all BSs use the same transmission power and have circular coverage. Then, we compute the probability that two BSs, *b*_*i*_ and *b*_*i*′_ are placed in the same group for the following two cases: Case 1) Either of *b*_*i*_ or *b*_*i*′_ is chosen as the pivot point; Case 2) Neither *b*_*i*_ nor *b*_*i*′_ is chosen as the pivot point.

#### Case 1)

Let *d*_*i*,*i*′_ be the distance between *b*_*i*_ and *b*_*i*′_. If *b*_*i*_ is chosen as one of *G* pivot points, then the other BS, *b*_*i*′_ must belong to the same group as *b*_*i*_. Thus, none of the *G* pivot points minus the chosen pivot *b*_*i*′_ can be located within a circle of radius *d*_*i*,*i*′_ centered at the BS *b*_*i*_ since the interferer’s transmission power is known to depend on the distance to the BS, *b*_*i*′_, as can be seen in Eq (2).

Let *f*_*M*,*G*_(*n*) be the probability of selecting *n* BSs among *M* BSs minus *G* pivot points. We then calculate the probability *f*_*M*,*G*_(*n*) as follows:

fM,G(n)=(M-Gn)(Mn)-1. (11)

Using Eq (11), the probability of selecting the BS *b*_*i*′_ given that *b*_*i*_ is the pivot point, *P*(*i*∣*i*′) is given by

$$\begin{array}{c} P\left(i^{\prime }|i\right)=\sum_{n=1}^{M-G-1}f_{M-2,G-1}\left(n\right)p_{i^{\prime }}\left(d_{i,i^{\prime }},n\right), \end{array}$$ (12)

where *p*_*i*′_(*d*_*i*,*i*′_, *n*) is the probability that a circle of radius *d*_*i*,*i*′_ centered around *b*_*i*′_ contains exactly *n* points, which is given by $p_{i^{\prime }}(d_{i,i^{\prime }},n)=\frac{{\left(\lambda \pi {\left(d_{i,i^{\prime }}\right)}^{2}\right)}^{n}e^{-\lambda \pi {\left(d_{i,i^{\prime }}\right)}^{2}}}{n!}$ following the PPP model. Then, in Case 1, we can derive the probability that two certain BSs, *b*_*i*_ and *b*_*i*′_, belong to a single group, $P_{c}^{\left(1\right)}$, as follows:

$$\begin{array}{ccc} P_{c}^{\left(1\right)} & = & \frac{G(M-G)}{M(M-1)}\left(P\left(i^{\prime }|i\right)+P\left(i|i^{\prime }\right)\right)\\ & = & \frac{2G(M-G)}{M(M-1)}P\left(i^{\prime }|i\right), \end{array}$$ (13)

where the first term, (*G*(*M*−*G*))/(*M*(*M* − 1)) indicates the probability of Case 1 occurring.

#### Case 2)

Consider an arbitrary BS and suppose that this BS is the *n*-th nearest one to the BS *b*_*i*_ (let’s say *b*_*i*′′_). We are now interested in obtaining the probability that the BS *b*_*i*_ belongs to the group represented by *b*_*i*′′_, which is denoted by *P*(*i*∣*i*′′). Note that the probability *P*(*i*∣*i*′′) is equivalent to the probability that the BS *b*_*i*′′_ becomes the nearest one of *b*_*i*_. Given the distance *d*_*i*,*i*′′_ from the BS, *b*_*i*_ to the *n*-th nearest BS, it follows that the probability *P*(*i*∣*i*′′) is given by

$$\begin{array}{c} P\left(i|i^{\prime \prime }\right)=\sum_{n=1}^{M-G-1}f_{M-1,G}\left(n-1\right)\frac{G}{M-n}{\hat{p}}_{i}\left(d_{i,i^{\prime \prime }},n\right), \end{array}$$ (14)

where ${\hat{p}}_{i}(d_{i,i^{\prime \prime }},n)$ denotes the probability density function (pdf) of *d*_*i*,*i*′′_, which is given by ${\hat{p}}_{i}(d_{i,i^{\prime \prime }},n)=\frac{2{\left(\lambda \pi \right)}^{n}{\left(d_{i,i^{\prime \prime }}\right)}^{2n-1}e^{-\lambda \pi {\left(d_{i,i^{\prime \prime }}\right)}^{2}}}{(n-1)!}$, using the PPP model.

We next derive the probability that the BS *b*_*i*′_ also selects *b*_*i*′′_ as its pivot point *P*(*i*′∣*i*′′), conditioned on the fact that the BS *b*_*i*_ belongs to the group represented by *b*_*i*′′_. Let *A*_*i*_(*r*) and *A*_*i*′_(*r*) denote circular areas with a radius of *r* centered around the BSs *b*_*i*_ and *b*_*i*′_, respectively. Then, we can express the probability *P*(*i*′∣*i*′′) as follows:

$$\begin{array}{c} P\left(i^{\prime }|i^{\prime \prime }\right)=\sum_{n=0}^{M-G-2}f_{M-3,G-1}\left(n\right){\tilde{p}}_{i^{\prime }}\left(D_{i^{\prime },i^{\prime \prime }},n\right), \end{array}$$ (15)

where ${\tilde{p}}_{i^{\prime }}(D_{i^{\prime },i^{\prime \prime }},n)$ is the probability that a subdomain $D_{i^{\prime },i^{\prime \prime }}=D\cap \{A_{i^{\prime }}(d_{i^{\prime },i^{\prime \prime }})-A_{i}(d_{i,i^{\prime \prime }})\}$ contains exactly *n* points.

Given the occurrence of Case 2, from Eqs (14) and (15), we get the probability that both *b*_*i*_ and *b*_*i*′_ belong to the group represented by a pivot point *b*_*i*′′_, $P_{c}^{\left(2\right)}$ as follows:

$$\begin{array}{c} P_{c}^{\left(2\right)}=\frac{\left(M-G)(M-G-1\right)}{M(M-1)}\underset{D}{\;\int \int \;}P\left(i|i^{\prime \prime }\right)P\left(i^{\prime }|i^{\prime \prime }\right)\frac{dA}{A(D)}, \end{array}$$ (16)

where the first term indicates the probability of Case 2 occurring.

Finally, from Eqs (13) and (16), we can derive the grouping probability in our proposed scheme as follows:

Pg=∑r=NkNc(Ncr)(Pc(1)+Pc(2))r(1-Pc(1)-Pc(2))Nc-r. (17)

### 5.2 Numerical Results

**Fig 2:**
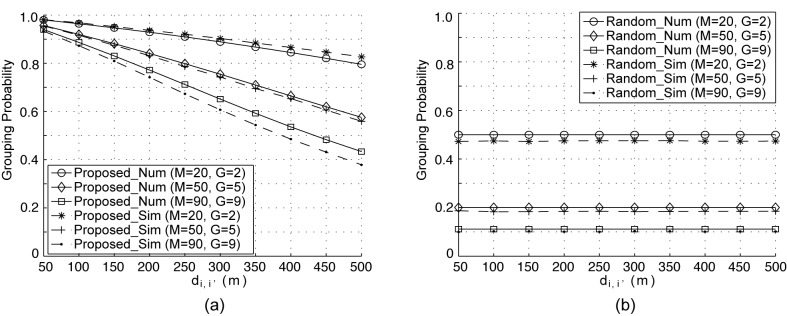
Grouping probability versus inter-cell distance per cycle for *N*_*c*_ = 1 and *N*_*k*_ = 1.

**Fig 3:**
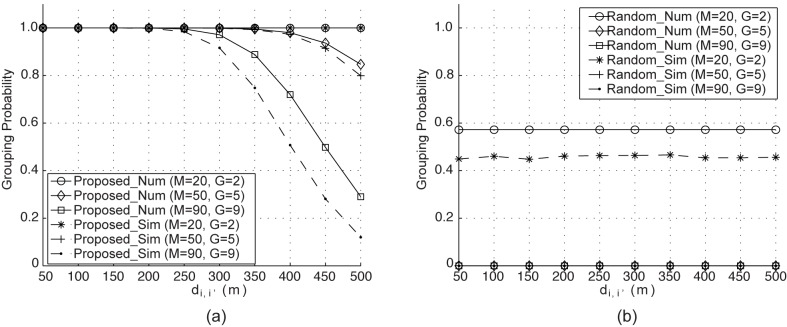
Grouping probability versus inter-cell distance per cycle for *N*_*c*_ = 30 and *N*_*k*_ = 15.

The results presented in [24] confirmed that their random-grouping approach performs better than EAs without grouping when tackling large optimization problems. Thus, in order to assess the accuracy of our analysis in terms of grouping probability, we carried out simulation tests for both the random and proposed grouping schemes, and compared the predictions of the analytical model from the previous section with these simulations. Figs 2 and 3 present both numerical and simulated grouping probabilities against *d*_*i*,*i*′_ for the cases of *N*_*c*_ = 1 and *N*_*c*_ = 30, respectively, under three different values of *M* and *G*. In Figs 2 and 3, ‘Num’ and ‘Sim’ indicate the numerical and simulated results, respectively. *D* is assumed to have a circular shape with a radius of 1,000 m. We examined the grouping behavior of the two schemes under heterogeneous inter-cell distances (*d*_*i*,*i*′_, 1 ≤ *i*, *i*′ ≤ *M*) ranging from from 50 m to 500 m, and in the simulation *M* BSs were placed randomly, with only the first two BSs set to be a distance of *d*_*i*,*i*′_ from each other. Each simulated data point was obtained by averaging the results of 10^5^ simulation runs.

It can be seen in Fig 2(a) that both the numerical and the simulated grouping probabilities decrease as *d*_*i*,*i*′_ increases in the proposed grouping scheme. This is because inter-cell interference decreases with an increase in the distance between the two BSs. On the other hand, as seen in Fig 2(b), all the curves for the random grouping are flat in the entire range of *d*_*i*,*i*′_ since the random grouping scheme does not consider inter-cell distance.

When producing the results for *N*_*c*_ = 30, *N*_*k*_ is set to 15, which indicates the probability that two BSs are assigned to one group for at least 15 cycles. The grouping probability in the proposed grouping decreases quickly when *d*_*i*,*i*′_ rises above 400 m and 300 m for *G* = 5 and 9, respectively, and the decrease for *G* = 9 is more rapid than that for *G* = 5. Even for *G* = 2, the grouping probability is flat regardless of the change in inter-cell distance. The reason for this phenomenon is that the smaller the number of groups, the lower the probability that two BSs belong to the same group. We also observe from Fig 3(b) that the grouping probability is the same regardless of the value of *d*_*i*,*i*′_ when random grouping is employed.

As can be seen in Figs 2 and 3, the predicted and the observed results correspond closely, with only a slight difference between the two.

### 5.3 Simulation Environment

**Fig 4:**
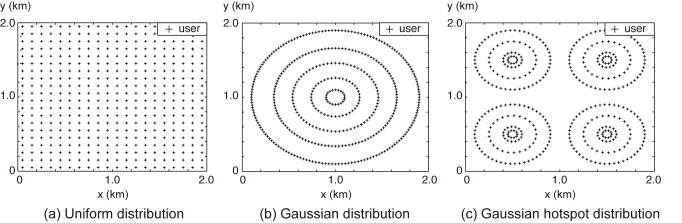
Different user distributions with 10 users/km (i.e., 400 users in the entire simulation area).

To evaluate the advantages of the proposed EA by means of simulation, we distributed users in a square area of 2 km × 2 km. To simulate an urban area with high user density [30], the user density was set to 10 users/km and 15 users/km, which are equivalent to 400 and 900 users, in the entire simulation area, respectively. Note that the user distribution affects the amount of traffic demand in an area, which should be considered when placing the BSs as presented in Section 3. To evaluate the effectiveness of the proposed EA in different user distributions, we used three user distribution models in the simulations: a uniform distribution, a Gaussian distribution, and a four-Gaussian hotspot distribution [4]. The three different user distributions are illustrated in Fig 4. In the uniform distribution, users are distributed uniformly over the entire area. The Gaussian distribution models a hot spot with user density at a maximum located at the center, and gradually decreasing toward the boundary. The four-Gaussian hotspot distribution models four hot spots with very densely located users. We omit the illustrations of the three distributions for the case of 15 users/km because, except for the density, they are the same as those shown in Fig 4.

All users were assumed to have the same traffic demand of 1 Mbps. With regard to the number of BSs, *M* was set to *N*/10, which is 10 users per BS. In the simulation, the value of the mutation rate is configured using *P*_*m*_ = 1/*substring*_*length*, where the substring length is 24 and the crossover rate is set to 0.9 [15]. As suggested in [23] and [31], the population size is set to 50. We set the maximum transmission power, *p*_*max*_, to 46 dBm [27, 30]. The simulation parameters are summarized in Table 1.

**Table 1:** Simulation parameters.

Parameter	Value
population size	50
crossover rate *P*_*c*_	0.9
mutation rate *P*_*m*_	1/24
number of groups *G*	*M*/10 groups
number of cycles *N*_*c*_	30 cycles
total number of users *N*	400 (10 users/km)
	900 (15 users/km)
maximum tx power *p*_max_	46 dBm

### 5.4 Simulation Results

As mentioned in Section 3, we started initially with *M* randomly located BSs, which will be referred to simply as ‘*M*-Random’ and then ran the proposed EA presented in Section 4 to find the optimal locations of the BSs. The simulation was performed in two scenarios, without and with macro BSs being installed initially. We first compare the performance of the proposed EA with that of *M*-Random to demonstrate that large gains cannot be achieved merely by installing more BSs.

**Fig 5:**
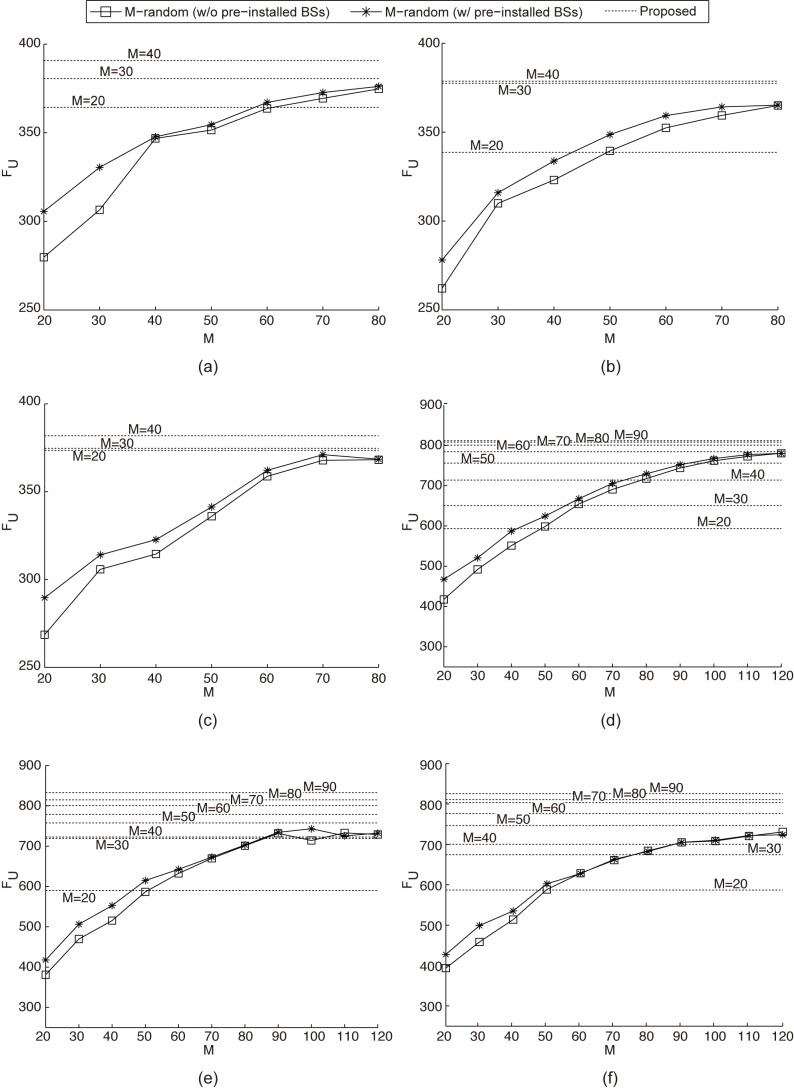
System throughput performance (a)-(c) when there are 10 users/km and (d)-(f) when there are 15 users/km.

Fig 5 shows the system throughput performance *F*_*U*_ of both *M*-Random and the proposed EA for each of the three user distributions. With *M* set to *N*/10, which are 40 and 90 for the two cases of 10 and 15 users/km, respectively, we simulate the proposed EA by increasing *M* by 10 from 20 to 40 and 90. For *M*-Random, *M* is increased from 20 to 80 and 120, respectively, when there are 10 and 15 users/km. In Fig 5, the horizontal lines indicate *F*_*U*_ in the BS deployment obtained by the proposed EA. For example, the horizontal line annotated as “M = 20” shows the system throughput of the proposed EA when starting the proposed EA with the *M*-Random for *M* = 20.

It is shown in Fig 5 that in *M*-Random, the slope of the increase tapers off even though the system throughput increases as *M* increases. This is because deploying too many BSs randomly can cause severe interference; that is to say, it is more likely for a user to suffer severe interference from a neighbor cell covering the same area as the serving cell of the user. Notably, the BS deployment that is obtained from the proposed EA starting with *M* = 40 (*M* = 90) achieves a system throughput almost as high as in *M*-Random with *M* = 80 (*M* = 120). That is, the proposed EA is able to achieve the same throughput by deploying a much smaller number of BSs than the random deployment approach. We can also see that the proposed EA shows a higher system throughput for the two Gaussian distributions than that for the uniform distribution, whereas the contrary phenomenon is observed in *M*-Random because the proposed EA finds the BS deployment that maximizes system throughput, as can be seen in Eq (7).

### 5.5 System Throughput Improvement

We now present the simulation results for the proposed EA, compared to the random-grouping based EA (RG-EA) [24–26] and an EA that detects interacting variables by monitoring the changes in the objective function and groups them as in [23]. We name the latter algorithm as EA with grouping based on interaction-detection (IDG-EA). When simulating IDG-EA, the threshold to identify the interacting variables is set to 10^−3^, as recommended in [23].

Because we aim to maximize the system throughput per user (i.e., *F*_*U*_) in this study, the system throughput improvement (denoted by Δ*F*_*U*_) is used as the main metric to evaluate the performance of the three algorithms. More specifically, Δ*F*_*U*_ indicates the amount of throughput improvement (in percentage) by the three algorithms over the initial random deployment of *M* BSs.

Tables 2–4 show the average, standard deviation, minimum and maximum of Δ*F*_*U*_ in RG-EA, IDG-EA, and the proposed EA for each of the three user distributions, without any macro BS being initially installed. Observe in Tables 2–4 that the proposed EA improves the performance of RG-EA by up to 18.97% and 20.68% for the two user densities of 10 and 15 users/km, respectively. Notably, when the uniform distribution is used and the user density is 10 users/km, the minimum value of Δ*F*_*U*_ in the proposed EA is even greater than that of RG-EA. We also observe that the statistics of Δ*F*_*U*_ in the proposed EA are higher than those in RG-EA for the other two user distributions. Specifically, Δ*F*_*U*_ of the proposed EA is up to 16.64% and 12.33% (17.08% and 12.52%) higher than that of RG-EA for the Gaussian distribution (the four-Gaussian hotspot distribution) when there are 10 and 15 users/km, respectively.

**Table 2:** System throughput improvement without any pre-installed macro BSs for the uniform distribution.

		RG-EA			IDG-EA			Proposed EA		
User Density	*M*	Avg.	Min.	Max.	Avg.	Min.	Max.	Avg.	Min.	Max.
10 (users/km)	20	40.75	37.78	42.97	31.33	25.63	39.63	48.48	47.27	50.04
	30	50.88	50.52	51.37	41.22	37.47	45.59	54.93	51.88	56.29
	40	28.55	27.41	29.75	22.20	21.79	23.34	32.15	30.99	33.54
15 (users/km)	20	87.26	83.28	90.17	61.57	47.12	72.30	105.31	102.73	107.84
	30	111.18	109.71	113.16	77.97	68.14	89.78	122.50	114.47	132.84
	40	119.13	114.95	124.40	97.23	89.06	107.07	124.13	121.38	125.59
	50	114.31	110.38	118.61	99.59	87.80	111.72	117.46	113.37	123.63
	60	99.23	96.45	102.13	79.50	77.11	82.37	105.63	100.22	109.80
	70	89.08	85.25	91.75	75.01	70.84	85.45	97.76	95.45	100.16
	80	84.89	81.35	87.33	69.24	61.51	77.63	85.95	84.30	87.88
	90	74.88	73.06	77.12	60.96	50.98	67.28	77.16	76.22	78.48

**Table 3:** System throughput improvement without any pre-installed macro BSs for the Gaussian distribution.

		RG-EA			IDG-EA			Proposed EA		
User Density	*M*	Avg.	Min.	Max.	Avg.	Min.	Max.	Avg.	Min.	Max.
10 (users/km)	20	51.89	50.01	56.03	34.90	31.65	40.53	60.52	53.64	65.19
	30	57.64	56.64	59.95	48.01	42.17	53.57	62.25	59.91	64.63
	40	42.56	41.48	43.54	33.03	27.95	38.14	45.02	43.39	47.31
15 (users/km)	20	154.92	147.09	160.76	118.54	108.40	137.65	170.84	165.22	177.03
	30	168.34	165.99	172.31	122.74	118.88	130.50	189.09	181.53	202.54
	40	183.87	175.69	188.35	141.99	118.71	161.44	197.50	186.71	206.43
	50	148.31	144.24	154.72	114.48	104.36	125.06	163.48	158.07	191.20
	60	128.11	120.93	131.55	107.58	91.20	116.21	138.41	128.36	149.71
	70	114.18	111.69	116.79	98.43	92.60	103.47	117.47	113.46	120.84
	80	104.98	102.22	109.23	78.44	64.87	88.24	108.30	102.89	112.75
	90	87.74	85.87	89.64	71.05	60.94	79.48	93.86	92.14	97.11

**Table 4:** System throughput improvement without any pre-installed macro BSs for the four-Gaussian hotspot distribution.

		RG-EA			IDG-EA			Proposed EA		
User Density	*M*	Avg.	Min.	Max.	Avg.	Min.	Max.	Avg.	Min.	Max.
10 (users/km)	20	60.04	57.63	64.31	34.37	29.67	39.16	70.29	62.51	74.09
	30	54.10	48.30	57.09	41.10	36.29	47.95	60.33	55.05	66.63
	40	52.61	51.92	53.26	44.03	40.01	49.95	56.37	55.80	56.92
15 (users/km)	20	128.12	120.42	138.06	74.14	63.31	81.58	144.15	140.94	147.68
	30	163.92	159.22	166.33	115.27	99.78	123.69	180.24	168.59	187.47
	40	164.69	158.12	169.18	120.82	107.00	146.33	171.79	164.99	180.47
	50	132.79	128.90	139.37	101.65	88.15	109.37	143.64	138.25	149.27
	60	123.42	121.16	126.38	97.35	92.59	103.36	130.27	123.82	133.34
	70	112.62	108.60	120.19	90.74	86.15	99.58	120.14	115.26	125.18
	80	111.32	109.07	112.84	88.22	81.91	100.52	116.44	115.96	117.20
	90	104.97	102.30	108.22	71.05	60.94	79.48	108.39	107.14	109.81

Tables 5–7 present the statistics of Δ*F*_*U*_ in the case that BSs are already installed in the simulation of an urban area in which some BSs are already installed such that inter-site distance (ISD) is 750 m [32]. For all three user distributions, the proposed EA shows a larger Δ*F*_*U*_ than that of RG-EA for user densities of both 10 and 15 users/km. Specifically, the average values of Δ*F*_*U*_ in the proposed EA are up to 11.78%, 16.28%, and 8.89% (10.89%, 11.57%, and 7.50%) higher than those of RG-EA for the uniform, the Gaussian, and the four-Gaussian hotspot user distributions, respectively, when the user density is 10 users/km (15 users/km).

**Table 5:** System throughput improvement for the uniform distribution when five macro BSs are already installed in the simulation area.

		RG-EA			IDG-EA			Proposed EA		
User Density	*M*	Avg.	Min.	Max.	Avg.	Min.	Max.	Avg.	Min.	Max.
10 (users/km)	20	50.89	50.45	51.32	41.42	39.98	42.65	56.89	54.53	58.75
	30	42.37	41.87	43.10	41.39	39.45	42.37	47.30	44.51	50.11
	40	39.30	38.21	40.96	36.51	34.97	38.02	41.14	39.75	43.04
15 (users/km)	20	116.63	111.30	121.38	97.54	95.06	100.92	123.52	118.31	125.89
	30	117.07	116.37	118.11	118.01	115.31	121.85	128.42	127.28	129.81
	40	118.89	115.70	121.71	112.72	108.93	118.48	124.15	119.90	126.91
	50	114.47	112.99	114.35	107.39	105.41	109.75	122.81	117.12	131.76
	60	109.34	108.75	110.13	80.67	70.09	86.90	111.64	109.33	116.83
	70	88.02	86.58	89.93	65.85	56.22	74.79	90.45	88.97	94.91
	80	74.10	71.42	78.18	58.47	54.31	61.97	76.89	75.47	78.68
	90	51.59	50.24	52.76	39.96	36.75	44.84	57.21	54.41	59.46

**Table 6:** System throughput improvement for the Gaussian distribution when five macro BSs are already installed in the simulation area.

		RG-EA			IDG-EA			Proposed EA		
User Density	*M*	Avg.	Min.	Max.	Avg.	Min.	Max.	Avg.	Min.	Max.
10 (users/km)	20	50.05	48.72	51.08	34.43	31.39	38.80	58.20	56.50	60.58
	30	57.02	55.23	58.94	50.44	46.91	53.41	58.80	56.18	62.59
	40	40.54	38.37	42.56	36.74	33.46	41.10	43.23	41.49	44.98
15 (users/km)	20	151.00	148.90	153.13	105.53	99.89	110.42	165.15	160.94	172.24
	30	187.66	184.37	193.76	110.24	101.58	118.06	209.38	204.18	214.03
	40	152.81	150.21	154.65	150.58	145.22	156.22	160.22	153.03	169.78
	50	134.39	130.14	137.68	120.33	116.99	128.03	138.42	131.13	142.80
	60	129.17	126.21	132.38	114.45	110.2	117.68	133.38	130.24	136.34
	70	120.22	114.96	125.47	97.45	88.29	107.66	123.32	117.78	127.83
	80	104.53	102.74	106.14	87.42	78.04	99.33	109.97	106.46	111.88
	90	90.59	87.80	91.91	79.63	74.94	82.79	94.50	91.92	98.44

**Table 7:** System throughput improvement for the four-Gaussian hotspot distribution when five macro BSs are already installed in the simulation area.

		RG-EA			IDG-EA			Proposed EA		
User Density	*M*	Avg.	Min.	Max.	Avg.	Min.	Max.	Avg.	Min.	Max.
10 (users/km)	20	77.13	71.67	79.95	60.10	58.93	62.15	79.56	75.77	84.89
	30	54.66	53.07	56.18	48.68	45.29	53.02	58.01	55.05	60.67
	40	52.39	45.00	54.08	47.57	45.44	50.74	57.05	53.94	59.16
15 (users/km)	20	143.03	140.17	144.90	109.21	91.73	129.88	153.59	148.85	159.40
	30	163.92	159.67	166.91	121.79	115.55	126.21	171.68	165.59	175.47
	40	152.70	149.28	156.53	130.78	117.23	144.09	159.90	157.32	164.99
	50	132.20	130.29	133.39	119.62	117.06	121.40	141.47	135.04	144.39
	60	130.66	127.07	132.64	114.80	108.72	121.41	140.45	134.10	148.50
	70	123.03	121.28	129.09	103.87	100.47	105.87	126.95	122.13	140.40
	80	121.00	116.93	122.99	101.33	85.54	103.63	123.84	119.49	128.47
	90	110.79	108.05	112.61	94.71	86.02	100.26	115.61	112.06	120.55

We see from the six tables that the proposed EA also achieves higher system throughput than IDG-EA. Specifically, when no macro BS is initially installed, the average values of Δ*F*_*U*_ in the proposed EA are up to 54.76%, 73.41%, and 104.47% (71.04%, 54.06%, and 94.42%) higher than IDG-EA for the uniform, Gaussian, and four hotspot user distribution, respectively, when the user density is 10 users/km (15 users/km). In the case that some BSs are installed, the average values of Δ*F*_*U*_ in the proposed EA are up to 37.34%, 69.02%, and 32.38% (43.18%, 89.84%, and 40.97%) higher than IDG-EA for the uniform, Gaussian, and four hotspot user distribution, respectively, when the user density is 10 users/km (15 users/km). We also observe that RG-EA outperforms IDG-EA as well. This is because in IDG-EA, more separable variables are classified as nonseparable ones.

Interestingly, we also find in the tables that maximum system throughput improvements are achieved for *M* = 20 or 30 when there are 10 users/km, while *M* = 30 or 40 when there are 15 users/km, and that after the point at which the maximum occurs, the throughput improvement starts to decrease. This is because installing more BSs causes harmful inter-cell interference, leading to a reduction in the system throughput improvement. Finally, we can say that the proposed EA outperforms both RG-EA and IDG-EA, not only for the uniform user distribution, but also for the other two practical user distributions.

## 6 Conclusion

In this paper, we have presented a correlation grouping approach for the application of an EA with the aim of designing an optimum network planning algorithm for large-scale LTE HetNets that results in the rapid convergence to optimal solutions. Noting that some HetNet cells have a strong correlation due to inter-cell interference, the correlation grouping approach makes variables with strong correlations (i.e., interfering cells) form groups, instead of grouping the individuals randomly when finding the optimal deployment of heterogeneous cells. We have also modified the variable-length genetic algorithm presented in [15] to be applied to the divided groups. To evaluate the performance of the proposed algorithm, we have analyzed the grouping probabilities and conducted simulations. Both numerical and simulation results confirm that the proposed algorithm outperforms both RG-EA and IDG-EA in terms of system throughput, not only for the uniform user distribution, but also for the practical Gaussian and four-Gaussian hotspot user distribution models.
