# Correction: Base Station Placement Algorithm for Large-Scale LTE Heterogeneous Networks

**DOI:** 10.1371/journal.pone.0143265

**Published:** 2015-11-12

**Authors:** The PLOS ONE Staff

There are errors in the Funding section. The correct funding information is as follows: This research was supported by Basic Science Research Program through the National Research Foundation of Korea (NRF) funded by the Ministry of Science, ICT & Future Planning (NRF-2013R1A1A3005914).

The publisher apologizes for the error.
